# Expression of SLP-2 Was Associated with Invasion of Esophageal Squamous Cell Carcinoma

**DOI:** 10.1371/journal.pone.0063890

**Published:** 2013-05-08

**Authors:** Wenfeng Cao, Bin Zhang, Fang Ding, Weiran Zhang, Baocun Sun, Zhihua Liu

**Affiliations:** 1 Key Laboratory of Cancer Prevention and Therapy, Department of Pathology, Tianjin Medical University Cancer Institute and Hospital, Tianjin, China; 2 State Key Laboratory of Molecular Oncology, Cancer Institute, Chinese Academy of Medical Sciences and Peking Union Medical College, Beijing, China; 3 State Key Laboratory of Breast Cancer Research, Department of Breast Cancer, Tianjin Medical University Cancer Institute and Hospital, Tianjin, China; Sun Yat-sen University Medical School, China

## Abstract

**Introduction:**

Stomatin-like protein 2 (SLP-2), a member of the Stomatin superfamily, has been identified as an oncogenic-related protein and found to be up-regulated in multi-cancers. Nonetheless, the expression pattern and regulation of SLP-2 in human esophageal squamous cell carcinoma (ESCC) remain unexplored.

**Methods:**

Immunohistochemistry and immunofluorescence staining analysis were performed to show SLP-2 expression and location. RNAi method was used to inhibit specific protein expression. Transwell assay was done to investigate cells invasive capability. RT-PCR and Western blot analysis were used to detect mRNA and protein expression levels.

**Results:**

Immunohistochemical analysis showed that up-regulation of SLP-2 was found in invasive front compared with cancer central tissue in ESCC. Inhibition of SLP-2 by SLP-2 siRNA can decrease ESCC cells invasive capability through MMP-2 dependent manner. Up-regulation of SLP-2 was effectively abrogated by the ERK1/2 inhibitors either PD98059 or U0126, but no effect was showed by the treatment of AKT inhibitors either LY294002 or MK-2206. So the regulation of SLP-2 was involved in activation of the MAPK/ERK pathway.

**Conclusions:**

We found that PMA/EGF could induce the up-regulated expression of SLP-2 probably through activating ERK signalling. The current study suggests that SLP-2 may represent an important molecular hallmark that is clinically relevant to the invasion of ESCC.

## Introduction

In the last decade, gene expression profiling of human cancer has proved valuable in cancer research, providing precious insight into mechanisms and targets involved in oncogenesis in several neoplasms [1]. By microarray analysis of various cancer tissues, we have led to the identification of multiple differentially expressed genes, which maybe serve as more important diagnostic or prognostic markers and even some new treatment targets for cancer.

Previously, we screened a series of genes with diverse expression in esophageal squamous cell carcinoma (ESCC) tissues as compared with their normal counterparts by complementary DNA (cDNA) microarray. Notably, one gene named human stomatin-like protein 2 (SLP-2) was dramatically highly expressed [2]–[4]. In 2000, the human SLP-2 sequence was first cloned and reported by Wang Y [5], and it is a novel and unusual member of the stomatin gene superfamily. Bioinformatics analysis showed that SLP-2 is highly conserved in development, its conservative stomatin-like domain was observed in 51 other proteins with potentially diverse functions. Based on its homology to stomatin, SLP-2 was predicted to be cytoplasmically located and also had the potential to be membrane-associated [6]. At present, SLP-2 gene’s structure and function remains less understood.

Aberrant expression of SLP-2 has been found in various malignancies [7]–[11]. Moreover, some recent evidences demonstrated SLP-2 may be associated with the character of cancer progression including invasion and metastasis [10]–[12], namely a significant correlation between SLP-2 high expression and the depth of ESCC invasion. But less is known about the accurate expression pattern in invasive tissues in esophageal cancer.

In current study, we analyzed the SLP-2 protein expression in different regions of invasive esophageal cancer tissue and generated SLP-2 depleted ESCC cells to examine the correlation and mechanism between SLP-2 and cancer cells invasion. We further explored whether the expression of SLP-2 can be regulated by the activation of MAPK/ERK and/or AKT pathway in human ESCC cell lines.

## Materials and Methods

### Tissue Specimens

Twenty fresh tumor tissues were taken immediately after surgery at the Tianjin Medical University Cancer Hospital from January to June 2008. Written informed consent was received from all participants before surgery. The above patients were diagnosed with ESCC by pathologist. The normal paired tissues were taken from the distal resection margins. Also, tumor center and tumor invasive margin tissues were collected, respectively. All specimens were stored at −80°C until the analysis. In addition, we still collected all of the above patients’ paraffin blocks to perform immunohistochemistry staining. None of the patients had received radiotherapy or chemotherapy before surgery. The project was approved by the Tianjin Medical University Cancer Institute and Hospital Research Ethics Committee.

### Cell Culture and Chemicals

ESCC cell line KYSE510 originally derived from primary human ESCC patients [13] was a kind gift from Dr. Y. Shimada, University of Kyoto. ESCC cell line EC9706 was from the tumor cell bank of Chinese Academy of Medical Sciences. The above two cell lines were grown in RPMI 1640 medium supplemented with 10% fetal bovine serum, 100 µg/µl streptomycin, and 100 µg/µl penicillin (pH 7.2–7.4) in a humidified incubator containing 5% CO_2_ at 37°C. PMA and EGF were purchased from Sigma-Aldrich (St Louis, MO). Two ERK1/2 inhibitors (PD98059 and U0126) and two AKT inhibitors (LY294002 and MK-2206) all were purchased from Selleck Chemicals LLC (Houston, TX), using at 1 µM.

### Immunohistochemistry Analysis

Immunohistochemistry staining was performed with the polyclonal primary antibody against SLP-2 at 1∶200 dilution (Proteintech Group Inc, Chicago, IL) as the method [9]–[10], [14]. Negative controls were conducted by replacing the primary antibody with phosphate-buffered saline. SLP-2 expression was evaluated by 2 independent pathologists (W.C. and B.S.). In this study, the SLP-2 staining index (SI) was ranked only according to the percentage of positive cell marked by yellow particles observed in tumor cytoplasm/cell plasma membrane. Samples in which <25% of cells showed positive staining were rated 1, those with 25% to 50% positive stained were rated 2, those with 51% to 75% positive stained were rated 3, those with >75% positive stained were rated 4. We defined that the expression of SLP-2 protein (SI≥1) was positive.

### Immunofluorescence and Confocal Laser Scanning Microscopy

Immunofluorescence analysis was employed to investigate the expression and localization of SLP-2 protein in esophageal cancer tissues. For the staining procedure, The tissue slide was dewaxed with xylene and rehydrated through gradient ethanol into water. After endogenous peroxidase activity was quenched with 3% H_2_O_2_ for 30 minutes, tissue slide was washed with PBS, permeabilized with 0.2% Triton X-100 in PBS buffer for 2 minutes at room temperature, then washed with PBS again, blocked with normal goat nonimmune serum for 60 minutes. After overnight incubation at 4°C with rabbit anti-SLP-2 immunoglobulin G (1∶200), tissue slide was rinsed three times with PBS and incubated at 4°C for 12 hours with mouse anti-α-actin immunoglobulin G (1∶200, Sigma, St Louis, MO). Then, washed and stained with 4′,6-diamidino-2-phenylindole (DAPI 1∶5000, Sigma, St Louis, MO) in the dark for 15 minutes. After washed, followed by 60 minutes of incubation with mixed secondary antibodies, FITC-conjugated goat anti-rabbit immunoglobulin G (1∶50, Molecular Probes, Eugene, OR) and tetramethylrhodamine isothiocyanate–conjugated goat anti-mouse immunoglobulin G (1∶50, Molecular Probes), tissue slide was mounted with Mowoil and examined with Leica TCS SP2 confocal microscope (Leica Microsystems, Wetzlar, Germany). Series of images were processed and analyzed with the accompanying software package.

### Transfection and RNA Interference

The 19-nucleotide siRNA duplexes used in this study were purchased from Qiagen (Valencia, CA, USA) and contained the following sequences: SLP-2 siRNA (sense): 3′- ACGUAUCUGAGAAAGUGGG-5′ and scrambled siRNA (sense): 3′-UGCACUGUGCAAGCCUCUU-5′. Cells were transfected with siRNA oligonucleotides using Lipofectamine 2000 (Invitrogen, Calsbad, CA) according to the manufacturer’s recommendations.

### RNA Isolation and Reverse Transcriptase-polymerase Chain Reaction (RT-PCR)

Total cellular RNA was extracted from cells using the TRIzol reagent (Invitrogen, Carlsbad, CA, USA). One microgram of total RNA was reverse-transcribed using the Transcriptase SuperScript II Preamplification System for First Strand cDNA kit (Invitrogen). The primers for detecting MMP-2 cDNA were: 5′-CAC TTT CCT GGG CAA CAA AT-3′ (forward), 5′-CTC CTG AAT GC CCT TGA TGT-3′ (reverse). The primers for detecting internal control β-actin were: 5′-CAG AGC AAG AGA GGC ATC C -3′ (forward) and 5′-CTG GGG TGT TGA AGG TCT C-3′ (reverse). After an initial denaturation at 95°C for 2 min, cycling conditions were as follows: 30 cycles: 95°C for 30 s, 60°C for 30 s, and 72°C for 60 s. PCR products were analyzed by agarose gel electrophoresis and stained with ethidium bromide. The band intensities were analyzed by densitometry using Kodak Molecular Imaging Software.

### Transwell Invasion Assay

For cells invasion assay, the filter membrane with 8 µm pore size (Corning, Acton, MA) was coated both sides by 300 µg/ml Matrigel in serum-free RPMI 1640. After air-dry and sterilization, 29 µl of the plain medium with serum was added to the lower chamber of Costar transwell (Corning). Following, the above transfected cells were trypsinized and resuspended in serum-free RPMI 1640 with the density of 1.2×10^6^/ml and seeded 50 µl in the upper chamber, then incubated at 37°C for 20 hours. Noninvading cells remaining on the upper surface of the filter were removed with a cotton swab and rinsed with PBS for several times, and the cells that appeared on the lower surface of the filter were fixed with methanol for more than 15 minutes at −20°C and then washed in PBS, stained with crystal purple and counted under a microscope.

### Western Blot

Total proteins were extracted from cultured cells and patient tissues separated by SDS–polyacrylamide gel electrophoresis (PAGE), and then transferred into nylon membrane. The blotted membrane was incubated with antibody and detected with chemiluminescence reagents as described previously [10]. Rabbit polyclonal antibody against SLP-2 was 1∶1000 dilution. ß-actin (Sigma, St Louis, MO) antibody acted as a control for equivalent protein loading.

### Statistical Analysis

All of the statistical analyses were carried out using the SPSS software version 16.0 (SPSS, Inc, Chicago, IL). The differences between variables were evaluated with χ2 testing. The results were considered to be significant at *P* value less than 0.05.

## Results

### Expression Patterns of SLP-2 Protein in Invasive ESCC

**Figure 1:**
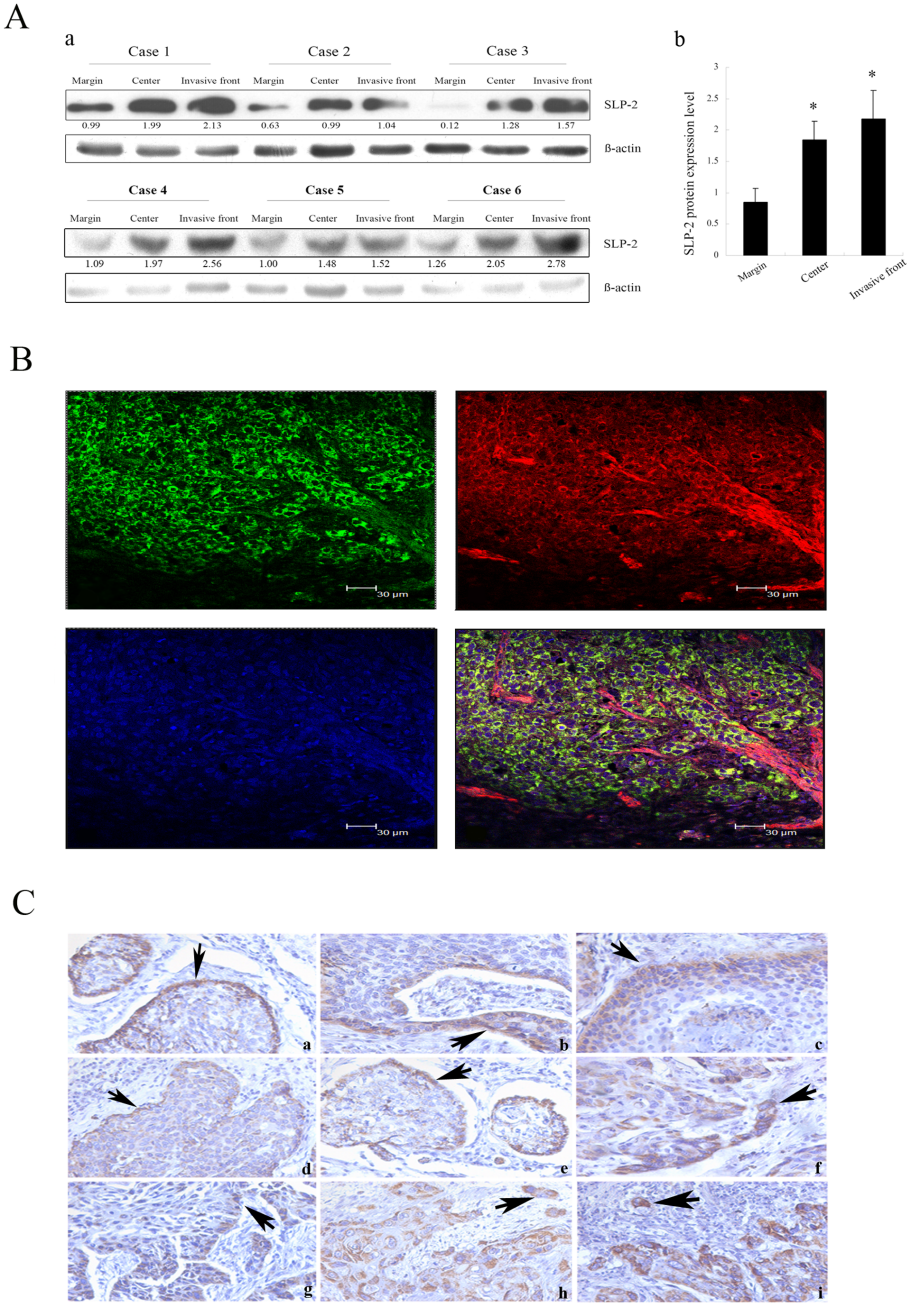
Expression patterns of SLP-2 protein in invasive ESCC. **Aa:** Overexpressions of SLP-2 in ESCC tissue than that in normal margin were detected by Western blot. Also, higher expression level of SLP-2 can be detected in invasive front tissues compared with that in tumor central tissues. ß-actin was used as a loading control. **Ab:** The graph represents densitometry of the results of 3 independent experiments (mean±SD). *, Statistically different, compared with the SLP-2 expression in normal margin (*P*<0.05). **B:** The distribution patterns of SLP-2 and cytoskeleton actin protein were observed by immunofluorescence staining. SLP-2 and actin protein were marked with green and red fluorescence, respectively. Merge image showed that ESCC cells infiltrated esophageal smooth muscles and SLP-2 protein expressed mainly at the invasive front. **Ca–g:** Predominant positive staining of SLP-2 at the invasive margin of cancer nests, which were indicated with black arrows. **Ch–i:** A single invasive cell, which was marked with black arrows showed SLP-2 staining (IHC staining, original magnification ×200).

In order to examine the expression patterns of SLP-2 in invasive ESCC tissues, we collected 20 paired of esophageal tumor central tissues, invasive margins and distal resection margins tissues and performed Western blot to detect the SLP-2 protein expression levels. The results showed that SLP-2 protein levels were higher in invasive front tissues than that in tumor central tissues (65%, 13/20). Also, SLP-2 levels were much higher in tumor central tissues than its expression in paired distal resection margins among 16 cases (80%) (Fig. 1Aa,b).

In order to show the distribution patterns of SLP-2 protein in ESCC tissues, double immunofluorescence stainings were performed. Esophageal smooth muscles and cytoskeleton proteins were marked by red fluorescence. Whereas, SLP-2 protein was stained with green fluorescence, which was basically assembled at the invasive margin. Under the confocal-microscope observation (shown in Fig. 1B), green signals of SLP-2 protein can completely merge red signals of cytoskeleton proteins α-actin forming uniform yellow fluorescence, which showed that SLP-2 protein and cytoskeleton protein can co-locate. In addition, IHC staining also showed SLP-2 expression pattern in ESCC tissues. In this study among 20 ESCC patients, SLP-2 positive expression were obviously observed at the invasive margin in 16 cases (Fig. 1Ca–g). Still some cases showed predominant positive staining of SLP-2 in cancer nests even a single invasive cell (Fig. 1Ch,i).

### SLP-2 Regulates Invasion of ESCC Cell Lines in vitro by Down-regulating MMP-2 Transcriptionally

**Figure 2:**
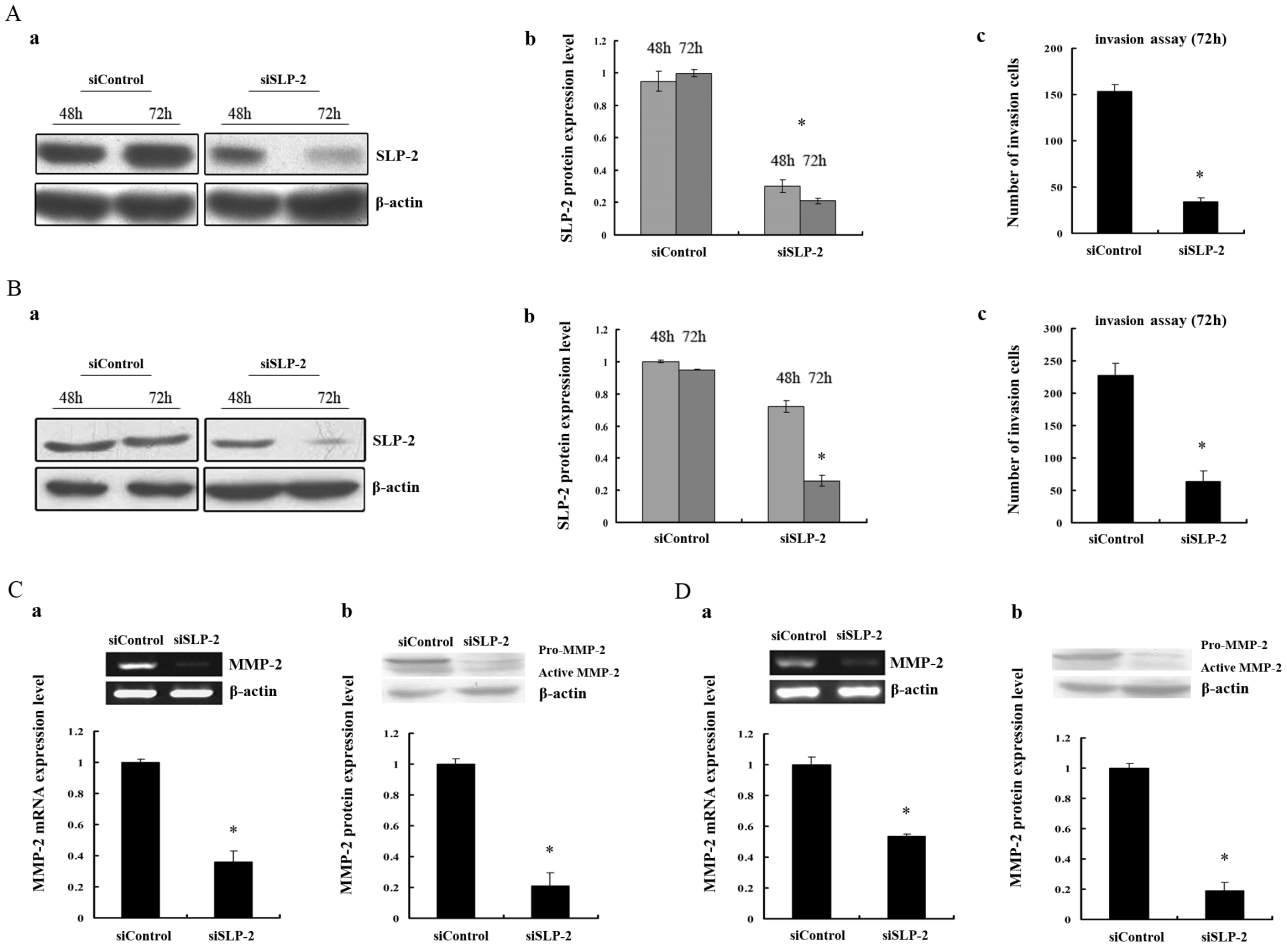
SLP-2 regulates invasion of ESCC cell lines in vitro by down-regulating MMP-2 transcriptionally. **Aa, Ba:** Human ESCC KYSE510 cells (A) and EC9706 cells (B) were transfected with either SLP-2 siRNA or control siRNA. After 48 h to 72 h, SLP-2 expressions were inhibited obviously, which were detected by Western blot. ß-actin was used as a loading control. **Ab, Bb:** The graph represents densitometry of the results of 3 independent experiments. *, Statistically different (*P*<0.05). **Ac, Bc:** The cells, which were transfected with either SLP-2 siRNA or control siRNA for 72 hours were transferred to Transwell chambers. After 20 hours, the cells were checked for invasion. *, Significant differences were observed between control and SLP-2 siRNA groups (*P*<0.05). **C, D:** KYSE510 cells (C) and EC9706 cells (D) were transfected with siRNA for control or SLP-2 for 72 hours, respectively. RT-PCR (Ca, Da) and Western blot assay (Cb, Db) were carried out. ß-actin was used as a loading control. The graph represents densitometry of the results of 3 independent experiments. *, Statistically different, compared with the control cells (*P*<0.05).

Due to the overexpression of SLP-2 at the invasion front in vivo and co-locate with cytoskeleton protein, we wondered whether SLP-2 overexpression can modify ESCC cells invasion in vitro. We investigated the invasive activity by an in vitro Boyden chamber assay. SLP-2 was antagonized by siRNA in vitro in two ESCC cells. Hence, the inhibitory effect of SLP-2 were evaluated by Western blot and the level of SLP-2 protein expression decreased more than 80% in KYSE510 cell and that of 70% in EC9706 cell after 72 hours (*P*<0.05, Fig. 2Aa,b, 2Ba,b). Seventy-two hours post-transfection, KYSE510 and EC9706 cells, which had been transfected with SLP-2 siRNA, showed a reduced invasion capacity of 78% and 70%, respectively compared with the cells having been transfected with scrambled siRNA (*P*<0.05, Fig. 2Ac, 2Bc).

MMPs are known to be crucial for degrading extracellular matrix components and for promoting tumor invasion. To further explore whether SLP-2 overexpression increasing ESCC cell invasive ability was due to the induction of MMPs, we used RT-PCR analysis revealed that the knockdown of SLP-2 in both KYSE510 and EC9706 cells drastically repressed MMP-2 expression at both mRNA level and protein level (*P*<0.05, Fig. 2Ca,b, 2Da,b). Taken together, the above results suggested that increase of cell invasive capacity by overexpression of SLP-2 is MMP-2 dependent.

### The MAPK Pathway and AKT Pathway Activation Might Probably be Correlated with SLP-2 Up-regulation

**Figure 3:**
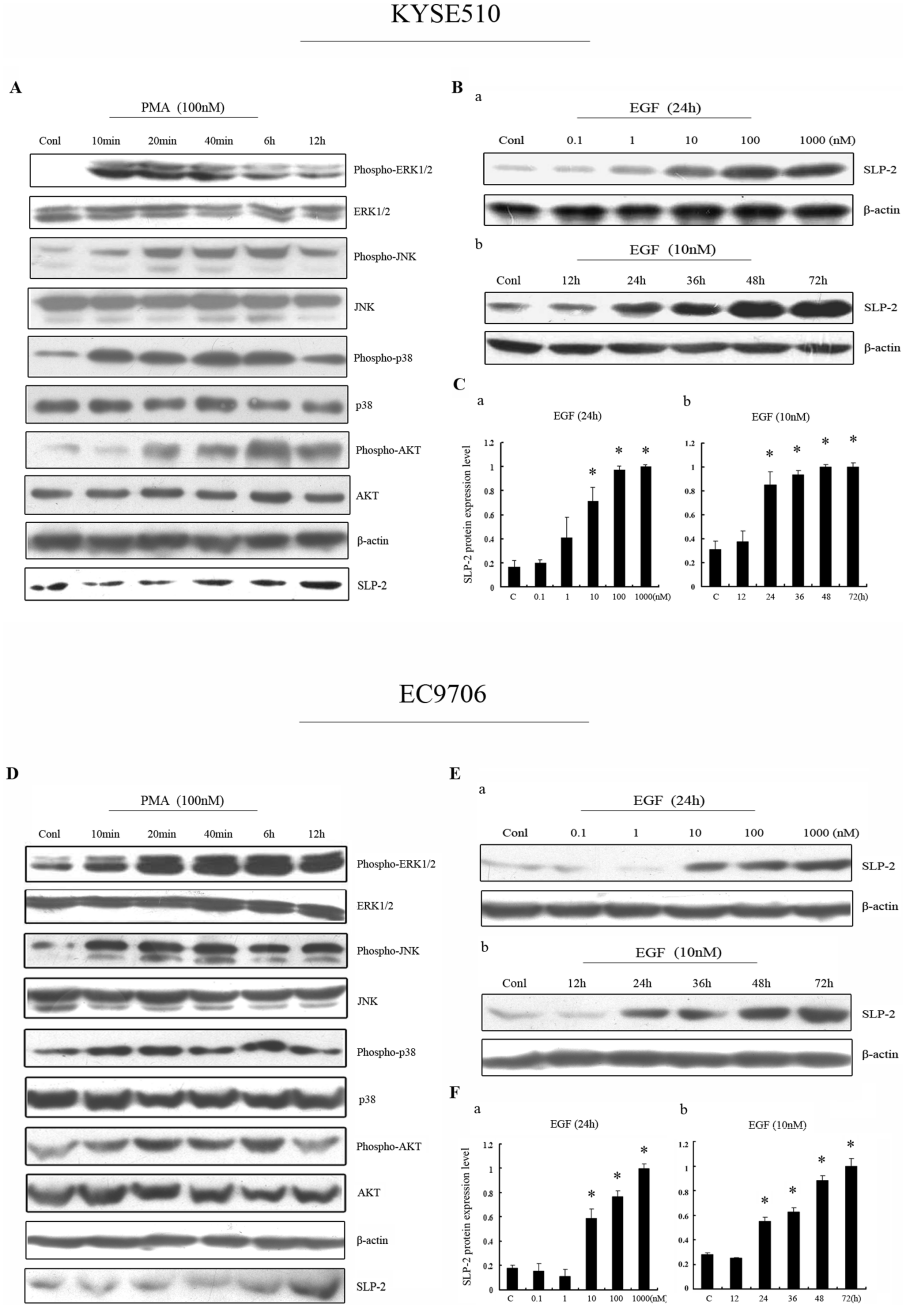
The MAPK pathway and AKT pathway activation might probably be correlated with SLP-2 up-regulation. **A, D:** In both KYSE510 cells (A) and EC9706 cells (D), PMA can activate MAPK/ERK, MAPK/JNK, MAPK/p38, and AKT pathway, induced p-ERK1/2, p-JNK, p-p38, and p-AKT high-expression. Whereas total ERK1/2, JNK, p38, and AKT expression remained unchanged. Also, followed by MAPK/AKT pathway activation, SLP-2 expression was raised obviously, which was identified by Western blot analysis. **Ba, b, Ea, b:** Similarly, in both KYSE510 cells (B) and EC9706 cells (E), EGF can up-regulate SLP-2 expression in both concentration and time dependent manners. **Ca, b, Fa, b:** KYSE510 cells (C), EC9706 cells (F). The graph represents densitometry of the results of 3 independent experiments (mean±SD). *, Statistically different, compared with the control cells (*P*<0.05).

To elucidate the signal pathway involved in the up-regulation of SLP-2, we investigated the activation of main signaling molecules by analyzing their phosphorylated forms with specific anti-phosphokinase antibodies in Western blot assay. Firstly we used PMA as a stimulator, the phosphorylation level of ERK1/2 in KYSE510 cell was elevated to the peak at 10 to 20 minutes. Then, gradually reduced to the low level after treatment for 12 hours, whereas the total amounts remained unchanged. Interestingly, followed by ERK pathway activation, SLP-2 expression was raised obviously at 12 hours identified by Western blot analysis (Fig. 3A). Moreover, we found that after stimulation by PMA, JNK, p38, and AKT pathway can be activated as well. In addition, EGF can activate the MAPK/ERK pathway and AKT pathway. Similar results showed that obvious up-regulation of SLP-2 by treatment with 10 nM EGF for 24 h, which suggested time and dose dependent manner in SLP-2 regulation (Fig. 3B, C). Simultaneously, similar results were obtained from another cell line EC9706. PMA can activate ERK, AKT. JNK and p38 pathway in very short time, and then after 12 hours SLP-2 expression was up-regulated correspondingly (Fig. 3D). Furthermore, EGF stimulation showed similar results (Fig. 3E, F).

### The Inhibitory Effects of Inhibitors Targeted MAPK/ERK Pathway can Down-regulate the SLP-2 Expression

**Figure 4:**
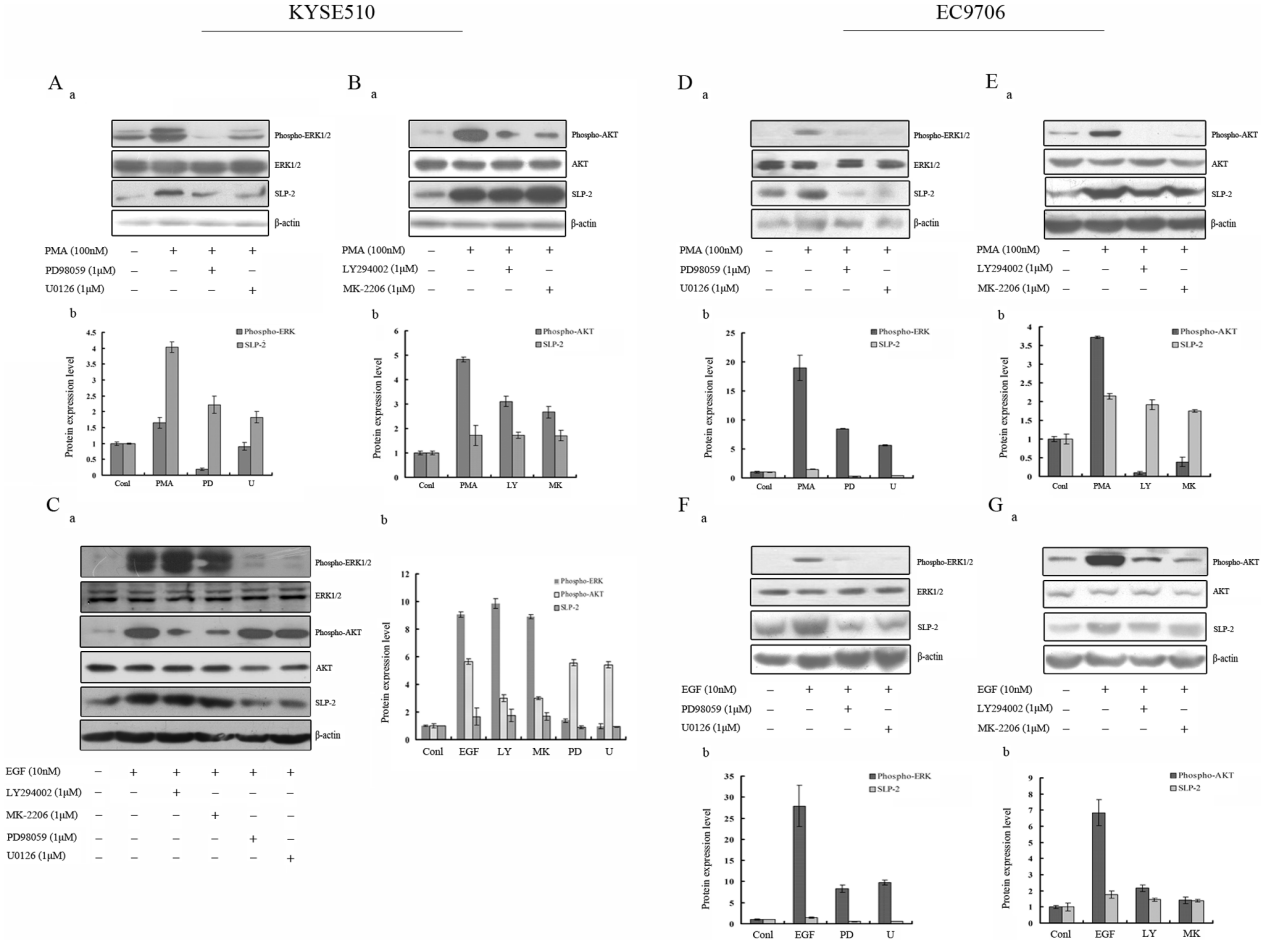
The inhibitory effects of inhibitors targeted MAPK/ERK pathway can down-regulate the SLP-2 expression. **Aa, Da:** Both KYSE510 cells (A) and EC9706 (D) were pretreated with two different ERK1/2 inhibitors (PD98059, 1 µM and U0126, 1 µM) for 3 hours, and then treated with PMA (100 nM) for 15 minutes to analyze the level of phosphorylated ERK1/2 and total ERK1/2. And then around 24 hours to detect SLP-2 expression. **Ab, Db:** The graph represents densitometry of the results of 3 independent experiments (mean±SD). **Ba, Ea:** Both KYSE510 cells (B) and EC9706 cells (E) were pretreated with two different AKT inhibitors (LY294002, 1 µM and MK-2206, 1 µM) for 3 hours, and then treated with PMA (100 nM) for 15 minutes to analyze the level of phosphorylated AKT and total AKT. Then, the rest of procedure of detection is similar. **Bb, Eb:** The graph represents densitometry of the results of 3 independent experiments (mean±SD). **Ca, Fa, Ga:** Both KYSE510 cells (C) and EC9706 cells (F, G) were pretreated with two different ERK1/2 inhibitors and two AKT inhibitors for 3 hours, and then treated with EGF (10 nM) for 15 minutes to analyze the level of phosphorylated ERK1/2, AKT and total ERK1/2, AKT. And then around 24 hours to detect SLP-2 expression. **Cb, Fb, Gb:** The graph represents densitometry of the results of 3 independent experiments (mean±SD).

To determine whether the MAPK/ERK and AKT pathways are involved in SLP-2 up-regulation, we examined the SLP-2 protein expression after selective MAPK/ERK or AKT inhibitors treatment. We pretreated KYSE510 cell with ERK1/2 inhibitors (PD98059 and U0126), and AKT inhibitors (LY294002 and MK-2206) respectively for 3 hours and then used PMA for 15 minutes to induce the activation of MAPK/ERK and AKT pathway. We found that either one of ERK1/2 inhibitors can effectively inhibit MAPK/ERK signal pathway activation by showing decreasing level of phosphorylated ERK1/2. Similarly, two inhibitors of AKT pathway both can block AKT pathway by down-regulation of phosphorylated AKT. And then, we revealed that after treatment with ERK1/2 inhibitors for 24 hours, the level of SLP-2 protein expression was decreased. Whereas no changes of SLP-2 expression were detected with AKT inhibitors treatment which were identified by Western blot analysis (Fig. 4A, B). Similar results were obtained after EGF stimulation (Fig. 4C). The above experiments were repeated by using another cell line EC9706. The results showed that after 24 hours inhibitors only targeted MAPK/ERK pathway can down-regulate the SLP-2 expression by either PMA or EGF stimulation (Fig. 4D–G).

## Discussion

Tumorigenesis is a complex and multistage process. Cancer invasion and metastasis are the final consequence of tumor progression and the most common cause of death in cancer patients [15]. Mechanisms for the acquisition of metastatic potential are, however, not well understood. Consequently, multiple factors and genes, that have been identifing, are involved in individual steps of invasion and metastasis. So, how can they act on invasion and what is the regulational mechanism of their expression also need to be widely investigated. Such findings might provide new clues for the novel approaches to overcome cancer invasion and metastasis.

Based on our previous work, the human SLP-2 could be regarded as a novel cancer-related gene. More importantly, high-level expression of SLP-2 protein had strong correlations with prognostic characteristics. In addition, recently we found that there existed a significant correlation between high-level SLP-2 protein expression and the invasive depth of ESCC. Namely, the deeper of the esophageal cancer invade, the higher expression of SLP-2 protein are [12]. In brief, SLP-2 high expression could act as an index of malignant biological behavior in cancer and correlate with tumor invasion and metastasis.

In this study, we found that 65% of the primary tumors had increased SLP-2 expression at the invasive margin tissues compared to the tumor centre tissues. In addition, under the confocal-microscope observation, we confirmed that most of SLP-2 protein basically expressed at the invasive front of cancer nests in ESCC tissue, even tried to infiltrate into surrounding esophageal smooth muscles. So elevated levels of SLP-2 should be an important factor possessing the aggressive behaviour in esophageal cancer cells. Recently, the biological processes at the invasive margin of tumors have been the scope of many studies [16]–[18]. Either the invasive front or the small clusters of malignant cells close to but separated from the invasive margin (tumor budding) was considered as the prognostic factor in the clinical setting. In our results, the elevated SLP-2 expression shown either at the invasive front or the presence of buddings was correlated to aggressive clinical outcome, indicating a process induced by the tumor cells to invade the surrounding tissues.

Tumor associated MMPs are quite important components in the invasive and metastatic process through their capacity to degrade extracellular matrix proteins. MMP-2 is one of the most vital proteins for degradation of the main constituent of the basement membrane and therefore involved in cancer invasion and metastasis [19]. Thus, in this study, we want to elucidate the MMP-2 expression mechanism to help understand the process of cell invasion. On the basis of previous results, we hypothesize that there exists a relationship between SLP-2 and MMP-2. So some of the invasive functions of SLP-2 might be mediated by up-regulation of MMP-2 expression. Therefore in this study, down-regulation of SLP-2 protein expression by transfected with SLP-2-specific siRNA in ESCC cell lines, KYSE510 and EC9706, resulted in decreased invasive ability as well as down-regulation of MMP-2 at both RNA and protein level. Together, our data illustrate the important role of SLP-2 in the regulation of MMP-2 expression as well as invasion of ESCC using in vitro cell models. So, until now we can understand a little about the significance of SLP-2 expression in ESCC, however the exact regulated mechanism of SLP-2 is obscure and need to be further studied.

Ras activation is a common intermediary in signaling pathways initiated by a variety of cell surface receptors, and signaling pathways downstream of Ras have been implicated repeatedly in oncogenesis. Signaling downstream of Ras is mediated by three major pathways, Raf/ERK, phosphatidylinositol 3 kinase (PI3K), and Ral guanine nucleotide exchange factor (RalGEF) [20]. Epidermal growth factor (EGF) and its receptor (EGFR) are frequently highly activated in placenta, and play pivotal roles in the regulation of proliferation. It has been reported that Raf/MEK/ERK and PI3K/AKT signaling pathways represent the downstream targets of activated EGFR. Activation of ERK pathway is often associated with oncogenic transformation. Phosphorylated ERK immunoreactivity has been related to tumour progression in oligodendrogliomas [21], and to metastasis in breast cancer [22]. In head and neck squamous carcinoma, the levels of activated ERK1/2 correlated with higher nodal status and a higher proliferation rate and were increased in tumour relapses [23]. Thus, in several cancers as well as in ESCC, ERK pathway activation is associated with tumorigenesis and tumor progression of esophageal squamous cell carcinoma [24]–[25].

Combining with the previous results, we found that SLP-2 was more important in ESCC invasion and metastasis, mainly expressing in the invasive front of cancer nests rather than that in the centre of tumors. In this study, we found PMA or EGF can activate ERK pathway by raising the level of phosphorylated ERK1/2. Moreover, ERK inhibitor can inhibit specific targeted molecular expression, deactivate ERK pathway, also down-regulate SLP-2 expression. In that case, the regulation of the expression of SLP-2 can be ERK pathway dependent. These findings indicated that SLP-2 may promote ESCC invasion through ERK signaling pathway, rather than PI3K/AKT signaling pathway. So, SLP-2 may be the downstream gene of the ERK pathway. Much work referring to the concrete regulation and other signaling pathway regulation of SLP-2 need to be further done.

Thus in ESCC, ERK pathway activation is associated with tumorigenesis and tumor progression, mainly including cancer invasion and metastasis. From our study, we can speculate the mechanism on how ESCC undergo invasion or metastasis. ERK pathway is activated quite frequently in ESCC. Afterwards, SLP-2 can be up-regulated by ERK pathway activation. Correspondingly, SLP-2 regulated MMP-2 high-expression. ESCC cells through MMP-2 dependent matrix degradation and regulation of cell adhesion moved through the ECM, break down the matrix and facilitate invasion.

In conclusion, data derived from this study have shown that the aberrant expression of SLP-2 might play important roles in the invasion of esophageal squamous cell carcinoma. SLP-2 down-regulation can inhibit ESCC cells invasion, which could be MMP-2 dependent manner. Also, the up-regulation of SLP-2 is involved in activation of the MAPK/ERK pathway.
